# Plant-Based Diet and Risk of Iron-deficiency Anemia. A Review of the Current Evidence and Implications for Preventive Strategies

**DOI:** 10.1007/s13668-025-00671-y

**Published:** 2025-06-18

**Authors:** Miguel López-Moreno, Adrián Castillo-García, Alberto Roldán-Ruiz, Isabel Viña, Gabriele Bertotti

**Affiliations:** 1 Diet, Planetary Health and Performance, Faculty of Health Sciences, Universidad Francisco de Vitoria, Pozuelo, Spain; 2 Systems Biology Department, University of Alcala, Madrid, Spain; 3 School of Physiotherapy, Faculty of Health Sciences, Universidad Francisco de Vitoria, Madrid, Spain; 4 Endocrinology and Nutrition Department, Hospital Universitari I Politecnic La Fe, Valencia, Spain

**Keywords:** Vegan diet, Vegetarian diet, Iron status, Anemia, Non-heme

## Abstract

**Purpose of Review:**

This review provides a comprehensive overview of iron metabolism, emphasizing the influence of dietary patterns—particularly vegetarian and vegan diets—on iron status and associated health outcomes.

**Recent Findings:**

Concerns regarding iron deficiency anemia in individuals following plant-based diets necessitate a deeper comprehension of the factors affecting iron bioavailability and absorption. Non-heme iron, which is more abundant in plant-based sources, poses challenges about its lower bioavailability and this could contribute to an increased risk of anemia in these populations. However, recent studies challenge this assumption, revealing a more complex relationship between plant-based nutrition and iron status. Additionally, emerging evidence suggests that the potential association between red meat consumption and cancer may be partially mediated by the high intake of heme iron.

**Summary:**

This review highlights the complex dynamics of dietary iron in vegetarian and vegan diets, which, despite offering less bioavailable iron, often surpass the intake levels of omnivorous diets. The potential involvement of adaptive physiological mechanisms suggests variability in non-heme iron absorption to meet nutritional requirements. While well-planned plant-based diets can be nutritionally adequate, further research is needed to better understand their long-term effects on iron metabolism.

## Introduction

Iron is part of the heme group of proteins, such as hemoglobin and myoglobin, which are involved in oxygen transport [1]. Also considered a hemoprotein, as catalases and peroxidases, iron is involved in oxygen metabolism and is part of cytochromes, a key component of cellular respiration. In addition, iron is found in nonheme iron proteins, including some subtypes of ribonucleotide reductase, which are essential in cellular processes such as deoxyribonucleic acid (DNA) synthesis and cell differentiation and proliferation [2, 3].

In recent years, there has been a surge in interest in plant-based diets, which are dietary patterns characterized by the exclusion of foods of animal origin. Among these plant-based diets, the vegetarian diet excludes meat and fish but may include dairy and eggs, while the vegan diet excludes all animal products, including meat, dairy, eggs, and honey [4]. In these diets, iron is found as non-heme iron, which can present challenges regarding efficient iron absorption [5]. This situation can lead to a higher risk of iron deficiency in vegetarian and vegan individuals, especially if these diets are not properly planned. Understanding how the bioavailability of iron and other nutrients affects health is crucial not only for ensuring optimal nutritional status in these individuals, but also for informing and improving dietary and public health recommendations.

This review aims to provide a comprehensive overview of the current understanding of iron metabolism, focusing on how dietary choices, including vegetarian and vegan diets, influence iron status and health outcomes.

### Regulation of Iron Status

An average adult requires an intake of approximately 20 mg of iron daily, predominantly for erythropoiesis—synthesis of erythrocytes in the bone marrow. Most of this demand is met through a continuous recycling process resulting from phagocytosis by macrophages of blood cells that have completed their average lifespan, and to a lesser extent, from food intake [6]. Approximately 1–2 mg of diet-acquired iron is needed to meet the remaining daily requirement of this trace element [7]. This amount is considerably lower than the dietary recommendations for iron, as the bioavailability of iron within food is limited. Heme (ferrous) iron is found in foods of animal origin and non-heme (ferric) iron in foods of plant origin, with an estimated bioavailability of 25–30% and 2–10%, respectively [8]. This bioavailability considerably fluctuates since intestinal absorption of iron is intricately regulated and influenced by various factors.

**Fig. 1:**
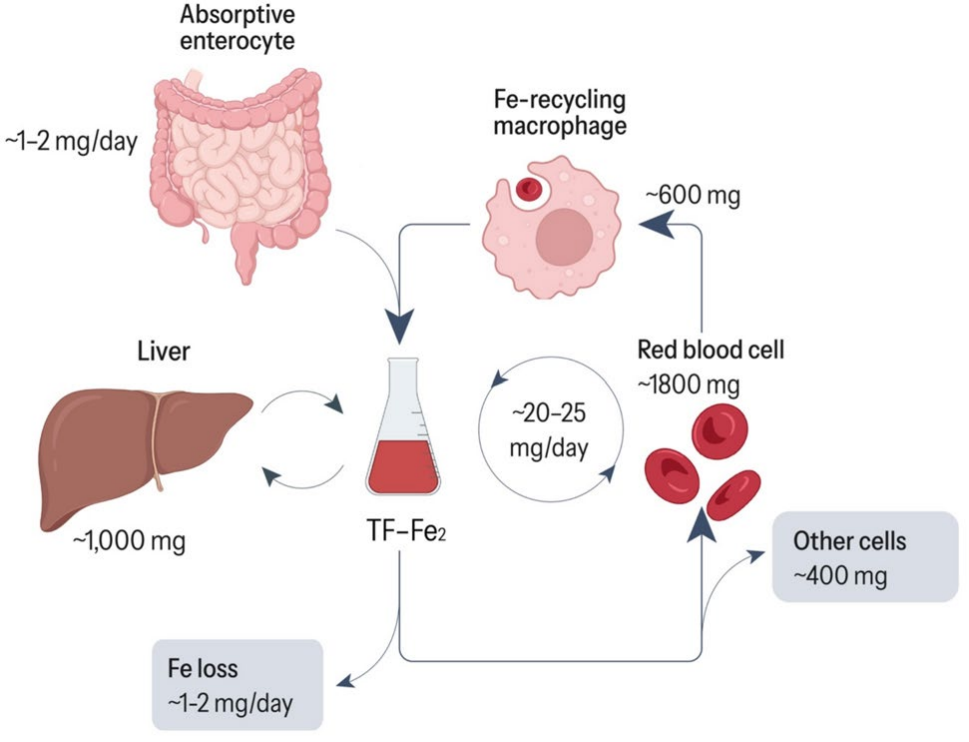
Systematic iron homeostasis Adapted from Galy, Conrad, and Muckenthaler [12]

In the intestine, dietary iron is absorbed in its heme form, thus non-heme iron (or Fe3 +) found in plant foods must first be reduced to heme iron (or Fe2 +) by duodenal cytochrome B reductase (DcytB) to facilitate the uptake into enterocytes by divalent metal transporter 1 (DMT1) [9]. Once inside the enterocyte, ferroportin enables its entry into the bloodstream from the intracellular [10]. Ceruloplasmin and hephaestin facilitate the oxidation of Fe2 + to Fe3 +, which is essential for its binding to transferrin, the primary iron transporter in the plasma [7]. Most of the absorbed iron is transported to the tissues that require this mineral, such as the bone marrow for red blood cell formation. Another fraction is used to maintain a stable serum iron pool, while the remainder is stored as ferritin-bound iron in tissues such as the liver or the spleen, after reduction to Fe2 +. The dietary iron is additional to that obtained from senescent red blood cells degraded by macrophages. All these processes are essential for proper body functioning (Fig. 1) [11].

Hepcidin is a hormone synthesised and secreted by hepatocytes that plays a crucial role in regulating iron balance. This hormone binds to ferroportin, leading to the internalisation and degradation of these iron transporters, thereby limiting the passage of iron into the blood [13]. When plasma iron levels are high, the body increases the production of hepcidin, which reduces the absorption of dietary iron in the intestines and the release of stored iron from tissues such as the liver. Conversely, when the concentration of iron in plasma decreases, the body triggers the opposite response. In pregnancy, plasma hepcidin concentration decreases as an adaptive mechanism to meet the increased iron requirements of this stage of life. This results in higher intestinal absorption of iron and increased mobilization of stored iron, along with elevated ferritin levels [14].

### Iron Deficiency Anemia

Anemia is a medical condition that reduces the number of functional red blood cells, which severely compromises their primary function of carrying oxygen throughout the body. This prevalent condition affects 24.3% of the global population, being especially relevant in women (31.2%) and children under 5 years old (41.4%) [15]. Iron deficiency anemia predominantly arises from substantial blood loss, which is attributable to gastrointestinal lesions from co-morbidities or significant menstrual bleeding. This might also be related to impaired dietary iron absorption, which is often due to gastrointestinal disorders like ulcerative colitis or coeliac disease [16]. Pregnancy is aphysiological state where iron demands escalate to support fetal development with sufficient oxygen and nutrients. In this situation, the risk of iron deficiency anemia surges and could be further exacerbated by an inadequate dietary iron intake [17]. The human body has compensatory mechanisms to regulate iron levels, such as increasing intestinal absorption or mobilizing stored iron; however, these adaptations may be insufficient in certain situations, leading to iron deficiency anemia.

### Major Interplay Between Anemia And Inflammation

**Fig. 2:**
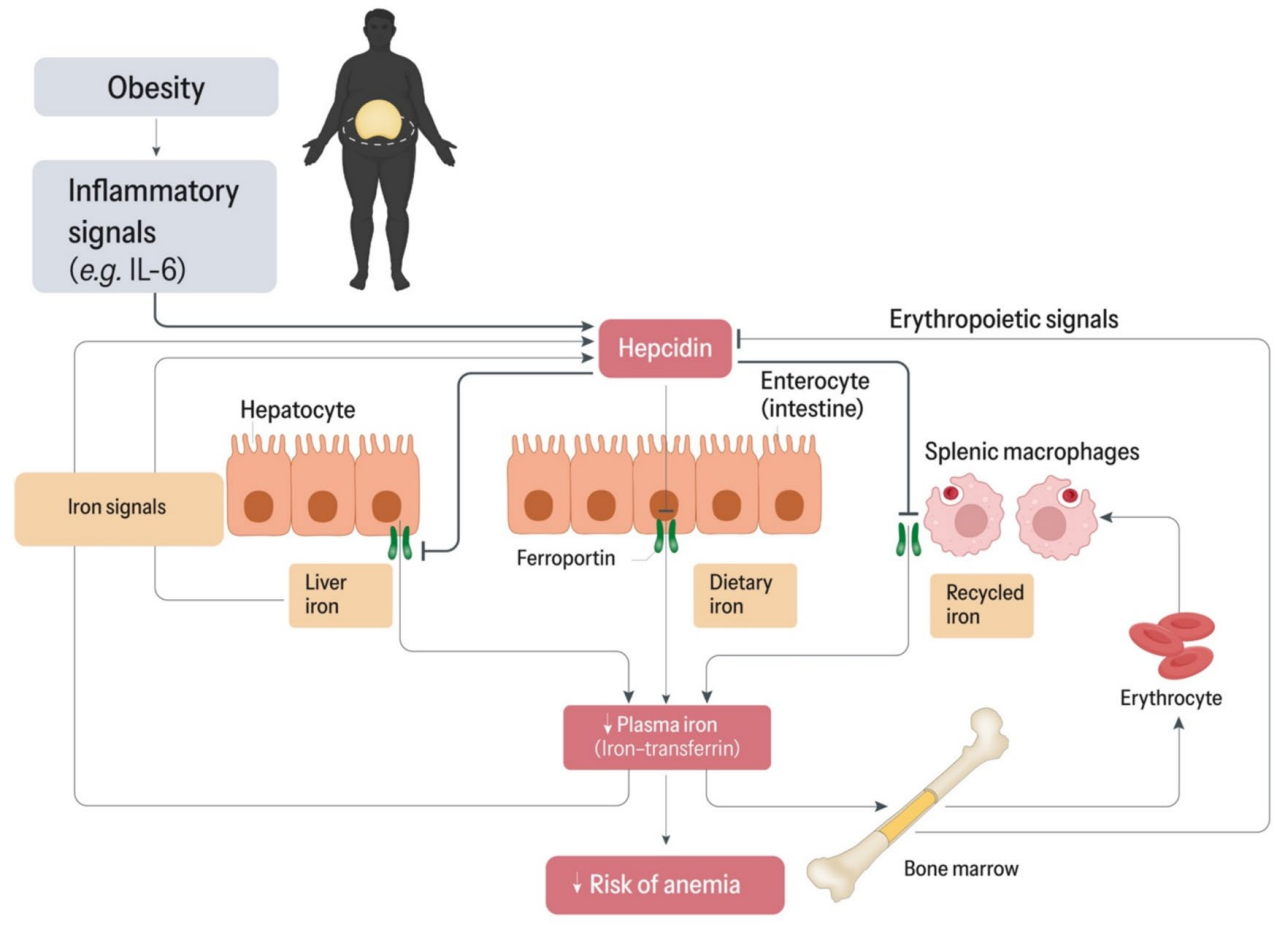
Role of chronic inflammation in iron homeostasis and anemia risk Adapted from Ganz & Nemeth [22]

Given that iron can influence pathogen virulence, its role extends beyond human physiology. When a pathogen enters the body, it triggers an inflammatory response mediated by macrophages releasing pro-inflammatory cytokines such as Interleukin-6 [18]. This leads to an increase in plasma hepcidin levels. Free plasma iron is stored in the body by binding to ferritin, thereby sequestering it [6]. It also limits the intestinal absorption of dietary iron, thus inhibiting the growth of pathogenic microorganisms. Overall, this mechanism is known as hypoferremia of inflammation, which is crucial from an evolutionary perspective in combating infections [19]. However, chronic inflammation may cause anemia of chronic disease due to a sustained increase in hepcidin levels, which limits the flow of plasma iron needed for erythropoiesis (Fig. 2). Moreover, the high prevalence of comorbidities associated with chronic inflammation contributes to an increase in disease burden [20, 21].

### Iron Uptake and Plant-Based Diet

The Institute of Medicine (IOM) recommends a higher iron intake for individuals following a vegetarian or vegan diet. Specifically, in these type of diets with limited consumption of animal foods, the recommended daily intake of iron is estimated at 14 mg for men and 32 mg for women of fertile age (compared to 8 mg and 18 mg for omnivorous diets, respectively) [23]. On the other hand, the World Health Organization/Food and Agriculture Organization of the United Nations (WHO/FAO) has established recommended nutrient intakes for iron bioavailability at 9.1 mg/day for adult males, 19.6 mg/day for women of childbearing age, and 7.5 mg/day for postmenopausal women [24]. Moreover, the European Food Safety Authority (EFSA) does not consider it necessary to establish dietary reference values for vegetarians as a separate population group, as the bioavailability of iron in European vegetarian diets does not significantly differ from that in diets containing meat and other flesh foods [25]. Assessing the adequacy of dietary iron intake is crucial for preventing conditions associated with its deficiency, such as iron deficiency anemia. The WHO considers hemoglobin values below 12 g/L for women and below 13 g/L for men as indicative of anemia [26].

Several studies have evaluated dietary iron intake and hemoglobin levels in vegan and omnivorous individuals. Sanders, Ellis, and Dickerson [27] analysed the hematological status of long-term vegans (20 males and 14 females) and found that none exhibited hemoglobin levels below the anemia threshold, indicating adequate iron status despite the absence of animal products in their diet.Likewise, a study from the United Kingdom (UK) Biobank cohort found that the prevalence of iron deficiency anemia did not differ between individuals with a vegan diet and those who consumed red or processed meat more than three times a week [28]. This was observed across all genders and age groups, including men, premenopausal women, and postmenopausal women. In contrast, women who followed a vegetarian diet (which may include some animal sources of iron) had a higher prevalence of anemia compared to those who consumed a regular meat diet [28]. In the Risks and Benefits of a Vegan Diet (RBVD) study, no differences were found between vegans and omnivores in any of the evaluated iron status markers [29]. In this study, iron intake was higher in vegans (22 mg/day) than in omnivores (14 mg/day), which may minimize the lower absorption of non-heme iron. A similar situation has been reported in a cohort of healthy German adults in which hemoglobin levels, mean corpuscular volume, mean corpuscular hemoglobin, ferritin, and transferrin showed no differences between vegans, lacto-ovo-vegetarians, and omnivores [30]. However, dietary iron intake was below recommendations in all cases (7.8 mg/day for omnivores, 9.2 mg/day for lacto-ovo-vegetarians, and 10.4 mg/day for vegans), and the use of iron supplements was more common among vegans (20% for omnivores, 30% for lacto-ovo-vegetarians and 48% for vegans). In addition, a recent observational study of 94 healthy adults found no higher prevalence of anemia in individuals following a vegan diet (with an average intake of 21.5 mg iron/day) compared to those following an omnivorous diet (with an average intake of 12.6 mg iron/day) [31]. Iron deficiency anemia in vegetarians and vegans may arise from insufficient energy intake, which limits the consumption of adequate dietary iron. Therefore, vegetarians and vegans must ensure sufficient energy intake to meet their iron needs [32, 33].

### Limitations of Ferritin In Plant-Based Diets

Regarding the diagnosis of iron deficiency anemia, ferritin is a commonly used marker due to its relation to iron stores [34]. It has been reported that vegetarians generally exhibit lower ferritin levels compared to the omnivorous population, indicating a lower content of stored iron and, therefore, an increased risk of iron deficiency anemia [35]. Ferritin levels typically increase in response to inflammation as a compensatory mechanism. A vegetarian or vegan diet is linked to lower levels of various inflammatory biomarkers, particularly C-reactive protein [36]. The higher consumption of fiber and antioxidants present in plant-based foods may justify this phenomenon, as it leads to multiple benefits including major chronic non-communicable diseases risk prevention. García-Maldonado et al., [37] found no significant differences in hemoglobin or mean corpuscular hemoglobin, but omnivorous participants had higher ferritin levels than vegans.This study did not evaluate inflammatory status, which could have influenced the results. In this regard, a recent study found that higher ferritin concentrations in omnivores compared to vegans were no longer significant after adjusting for overweight/obesity and elevated C-reactive protein levels [38].

### Dietary Sources of Iron

Given that iron is a crucial nutrient, dietary iron intake is essential to meet the body's needs. Iron within food is present in two forms: heme iron and non-heme iron. Heme iron is mainly found in animal-based foods such as offal, red meat, and bivalve molluscs, and has a higher bioavailability than non-heme iron, which is present in plant-based foods such as legumes, nuts, and seeds. The lower bioavailability of iron in plant sources has been attributed to the presence of certain components known as'anti-nutrients', such as phytates, oxalic acid and polyphenols [39]. These compounds can form complexes with iron, hindering their absorption in the intestine [8]. These processes are complex as some compounds (e.g., phytates) can induce physiological adaptations that increase intestinal iron absorption, thereby mitigating the reduced bioavailability of dietary iron [40, 41]. This may be related to lower levels of hepcidin, as reported in vegetarian children, suggesting increased intestinal absorption of dietary iron as a compensatory response [42]. Furthermore, the capacity of some'anti-nutrients'to form complexes with certain compounds is of great interest in other physiological contexts. These compounds can bind to potential carcinogens, counteracting their activity, or to free radicals, attenuating the formation of advanced glycation end products linked to the cellular ageing process [43].

It is important to note that the cooking method has a significant impact on the phytate content of food. For instance, soaking black beans (Phaseolus vulgaris L.) can reduce phytate content by 18%, which increases to 35% when subsequently cooked [44]. This decrease is more pronounced with longer soaking times and higher temperatures (50 °C compared to 30 °C), as these conditions promote phytase activity [45]. The phytate content in lentils decreases after dehulling (0.13%), sprouting (0.15%), and cooking (0.18%) [46]. Similarly, fermentation is an effective method for reducing the phytic acid levels in legumes such as soybeans or cowpeas. The longer the fermentation time, the greater the reduction [47]. In the same manner, bread produced through a long fermentation process exhibits a reduction of > 50% in phytate content when compared to raw flour [48]. This could be attributed to the decrease in pH, which promotes the hydrolysis of phytic acid by phytases [49].

**Fig. 3:**
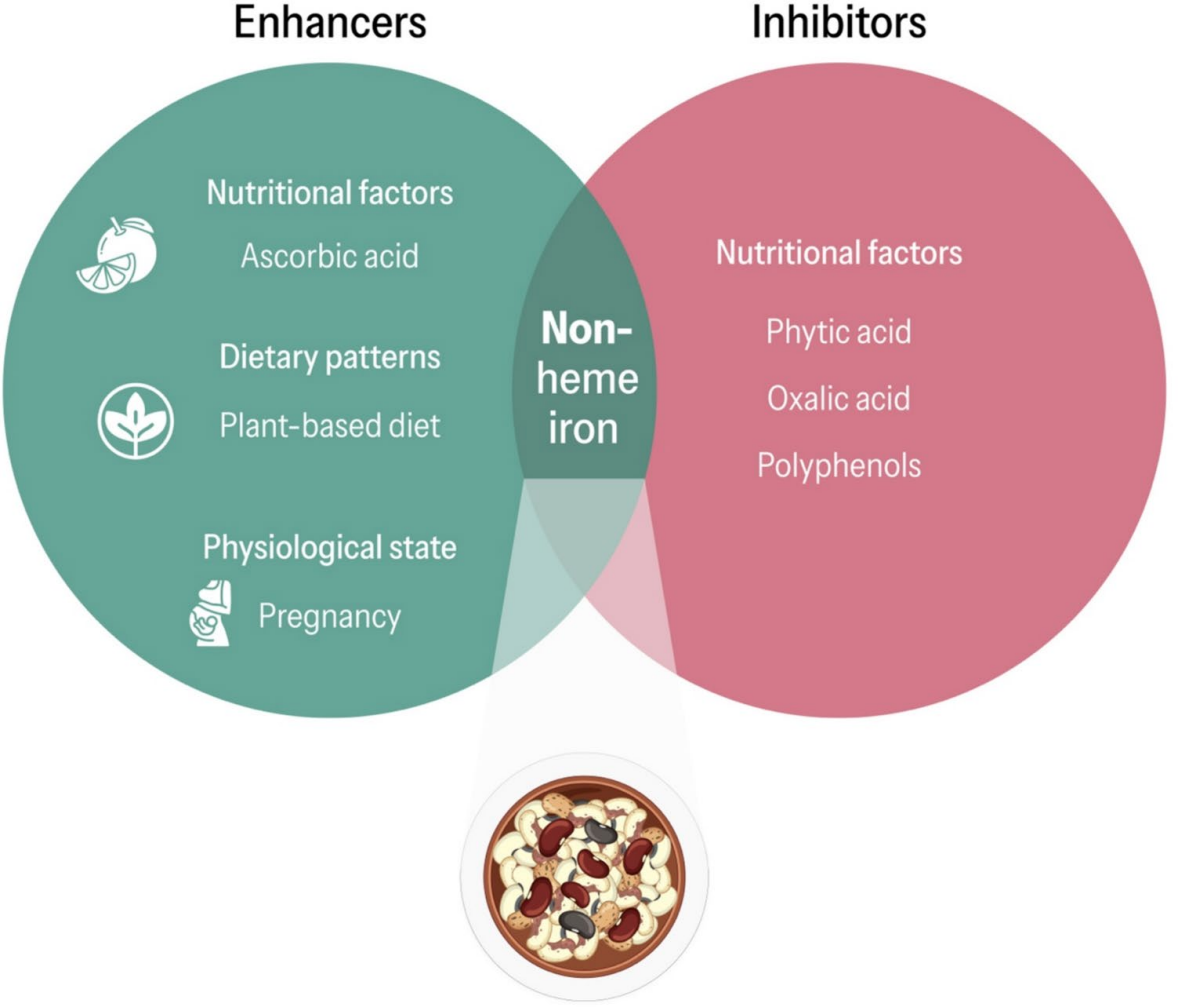
Factors regulating the bioavailability of non-heme iron

Certain compounds found in vegetables, such as vitamin C or ascorbic acid, are particularly effective in enhancing the bioavailability of dietary iron. In the gastrointestinal tract, this antioxidant inhibits oxidation of Fe^2+^, which is the only form of dietary iron absorbed in the gut [50]. In 1980, Sean R. Lynch and Jame D. Cook discovered that ascorbic acid forms complexes with Fe3 + (non-heme iron) at acidic pH. This complex remains soluble in the intestine and encourages its conversion to Fe^2+^, which subsequently passes to the enterocyte [51]. The absorption of dietary iron is dependent on the dose of vitamin C and requires co-administration [52]. In a previous study conducted by Siegenberg et al. [53], it was observed that the absorption of iron in 80 g of bread increased gradually from 6.7% to 12.6% when combined with 30 mg of vitamin C, from 3.8% to 10.4% with 50 mg of vitamin C, and from 10.4% to 27.4% with 150 mg of vitamin C. Due to the thermolabile nature of vitamin C, it is advisable to prioritize cooking techniques that do not cause a reduction in vitamin C content, such as raw, steamed, or microwaved [54] (Fig. 3).

## Adverse Effects of Iron

Despite iron's essential role in nutrition, it can be deleterious under specific conditions. The'Fenton reaction', discovered by Henry John Horstman Fenton in 1894, illustrates this phenomenon. In this reaction, ferrous iron (Fe2 +) interacts with hydrogen peroxide (H2O2) to produce highly reactive hydroxyl radicals (-OH) [55]. Free radicals play important physiological roles in the body including cellular signalling pathways and immune regulation [56]. However, an imbalance between their production and the availability of antioxidant compounds leads to oxidative stress, which is associated with many chronic non-communicable diseases [57]. The Fenton reaction can lead to the overproduction of free radicals, which can damage genetic material and cause lipid peroxidation; that is the degradation of lipids in cell membranes [58]. This situation triggers an iron-dependent cell death process called ferroptosis, which has recently been identified and may be involved in several neurological conditions [59].

The International Agency for Research on Cancer (IARC) classified red meat as a probable human carcinogen (Group 2 A) and processed meat as a human carcinogen (Group 1) [60]. Research suggests that a link between cancer and red meat consumption may be influenced by the impact of consuming high levels of heme iron. Bastide et al., [61] conducted a meta-analysis which found that individuals with a higher intake of heme iron had an 18% greater risk of colon cancer compared to those with a lower intake. This may explain the risk of colon cancer being higher when consuming beef, as this food has a higher heme iron intake compared to other types of red meat [62]. Similarly, the National Institutes of Health-American Association of Retired Persons (NIH-AARP) Diet and Health Study found a positive correlation between the intake of heme iron and the risk of breast cancer [63].

Likewise, this association has also been reported in other types of cancer, including endometrial, pancreatic, and lung cancer [64–66]. However, this link has not been identified with non-heme iron. One recent study substituted heme with non-heme iron, which led to a lower risk of colorectal cancer in men (HR:0.94; 95%CI: 0.89, 0.99) [67]. Various mechanisms have been proposed to explain this association: for instance, excessive heme iron intake can cause iron overload, leading to the production of free radicals through the Fenton reaction. This can result in DNA adducts and lipid peroxidation [68]. Similarly, heme iron acts as a catalyst for the formation of nitrosamines (NOCs), which are recognised carcinogens according to the IARC [69]. Furthermore, the consumption of red meat may lead to the displacement of other plant-origin protein sources, such as legumes and whole grains (Gardner et al. [70]; López-Moreno [71]). These foods are high in fiber and polyphenols, which have been shown to exert a protective effect against various types of cancer, including colorectal cancer [72, 73]. The fermentation of fiber and polyphenols by gut bacteria produces short-chain fatty acids (SCFA), which can reduce the formation of secondary bile acids such as deoxycholic acid and lithocholic acid. These secondary bile acids pose a cytotoxic risk to colonocytes [74]. In addition, butyrate is a SCFA that promotes histone acetylation. This leads to the inhibition of cell proliferation and apoptosis [75].

## Conclusion

This review explores the complex balance of dietary iron in vegetarian and vegan populations, highlighting a potential paradox where plant-based diets, traditionally considered lower in bioavailable iron, may induce physiological adaptations that enhance its bioavailability to maintain adequate iron status. Furthermore, the convergence of sufficient caloric intake with a higher dietary iron intake provides a compelling argument for the nutritional adequacy of plant-based diets. However, the complexities of iron bioavailability and its systemic effects warrant further investigation.

## Key References

- Slywitch E, Savalli C, Duarte ACG, Escrivão MAMS. Iron Deficiency in Vegetarian and Omnivorous Individuals: Analysis of 1340 Individuals. Nutrients. 2021;13.
  - ○ This study provides an in-depth analysis of how nutritional status and inflammation levels influence ferritin levels, potentially complicating the accurate diagnosis of iron deficiency in both vegetarian and omnivorous individuals. It highlights that, compared to vegetarians, women who do not menstruate and men exhibit the same prevalence of iron deficiency when following an omnivorous diet
- Armah SM, Boy E, Chen D, Candal P, Reddy MB. Regular Consumption of a High-Phytate Diet Reduces the Inhibitory Effect of Phytate on Nonheme-Iron Absorption in Women with Suboptimal Iron Stores. J Nutr. 2015;145:1735–9.
  - ○ This randomized controlled trial investigates whether habitual consumption of a high- phytate diet reduces the inhibitory effect of phytate on non-heme iron absorption among young women with suboptimal iron stores. The trial examines the potential role of dietary protein in enhancing iron bioavailability in this population.
- Hunt JR, Roughead ZK. Adaptation of iron absorption in men consuming diets with high or low iron bioavailability. Am J Clin Nutr. 2000;71:94–102.
  - ○ This study examines how iron absorption adapts in men fed diets with high or low iron bioavailability, adjusting homeostatically to maintain body iron stores.

## Data Availability

No datasets were generated or analysed during the current study.

## References

[1] Winter WE, Bazydlo LAL, Harris NS. The molecular biology of human iron metabolism. Lab Med. 2014;45:92–102. 24868988 10.1309/lmf28s2gimxnwhmm

[2] Pantopoulos K, Porwal SK, Tartakoff A, Devireddy L. Mechanisms of Mammalian Iron Homeostasis. Biochemistry. 2012;51:5705–24. 22703180 10.1021/bi300752r PMC3572738

[3] Sanvisens N, Bañó MC, Huang M, Puig S. Regulation of ribonucleotide reductase in response to iron deficiency. Mol Cell. 2011;44:759–69. 22152479 10.1016/j.molcel.2011.09.021 PMC3240860

[4] Hargreaves SM, Rosenfeld DL, Moreira AVB, Zandonadi RP. Plant-based and vegetarian diets: an overview and definition of these dietary patterns. Eur J Nutr [Internet]. 2023;62:1109–21. 10.1007/s00394-023-03086-z. 36681744 10.1007/s00394-023-03086-z

[5] Saunders A, Craig W, Baines S, Posen J. Iron and vegetarian diets. Med J Aust [Internet]. 2013 [cited 2024 Jul 1];199:S11–6. Available from: https://pubmed.ncbi.nlm.nih.gov/25369923/10.5694/mja11.11494 25369923

[6] Wang C-Y, Babitt JL. Liver iron sensing and body iron homeostasis. Blood. 2019;133:18–29. 30401708 10.1182/blood-2018-06-815894 PMC6318427

[7] Vogt A-CS, Arsiwala T, Mohsen M, Vogel M, Manolova V, Bachmann MF. On iron metabolism and its regulation. Int J Mol Sci. 2021; 27;22(9):4591. 10.3390/ijms22094591 PMC8123811 33925597

[8] Piskin E, Cianciosi D, Gulec S, Tomas M, Capanoglu E. Iron Absorption: Factors, Limitations, and Improvement Methods. ACS Omega. 2022;7:20441–56. 35755397 10.1021/acsomega.2c01833 PMC9219084

[9] Knutson MD. Iron transport proteins: Gateways of cellular and systemic iron homeostasis. J Biol Chem. 2017;292:12735–43. 28615441 10.1074/jbc.R117.786632 PMC5546014

[10] Roemhild K, von Maltzahn F, Weiskirchen R, Knüchel R, von Stillfried S, Lammers T. Iron metabolism: pathophysiology and pharmacology. Trends Pharmacol Sci. 2021;42:640–56. 34090703 10.1016/j.tips.2021.05.001 PMC7611894

[11] Sandnes M, Ulvik RJ, Vorland M, Reikvam H. Hyperferritinemia-A Clinical Overview. J Clin Med. 2021;10(9):2008. 10.3390/jcm10092008 PMC8125175 34067164

[12] Galy B, Conrad M, Muckenthaler M. Mechanisms controlling cellular and systemic iron homeostasis. Nat Rev Mol Cell Biol. 2024;25:133–55. 37783783 10.1038/s41580-023-00648-1

[13] Sangkhae V, Nemeth E. Regulation of the Iron Homeostatic Hormone Hepcidin. Adv Nutr. 2017;8:126–36. 28096133 10.3945/an.116.013961 PMC5227985

[14] Fisher AL, Nemeth E. Iron homeostasis during pregnancy†‡. Am J Clin Nutr. 2017;106:1567S-1574S. 29070542 10.3945/ajcn.117.155812 PMC5701706

[15] Gardner WM, Razo C, McHugh TA, Hagins H, Vilchis-Tella VM, Hennessy C, et al. Prevalence, years lived with disability, and trends in anaemia burden by severity and cause, 1990–2021: findings from the Global Burden of Disease Study 2021. Lancet Haematol. 2023;10:e713–34. 37536353 10.1016/S2352-3026(23)00160-6 PMC10465717

[16] Cappellini MD, Musallam KM, Taher AT. Iron deficiency anaemia revisited. J Intern Med. 2020;287:153–70. 31665543 10.1111/joim.13004

[17] DeLoughery TG. Iron Deficiency Anemia. Med Clin North Am. 2017;101:319–32. 28189173 10.1016/j.mcna.2016.09.004

[18] Nairz M, Weiss G. Iron in infection and immunity. Mol Aspects Med. 2020;75:100864. 32461004 10.1016/j.mam.2020.100864

[19] Ganz T, Nemeth E. Hypoferremia of inflammation: innate host defense against infections. Blood Cells Mol Dis. 2024;104:102777. 37391347 10.1016/j.bcmd.2023.102777

[20] Wiciński M, Liczner G, Cadelski K, Kołnierzak T, Nowaczewska M, Malinowski B. Anemia of Chronic Diseases: Wider Diagnostics-Better Treatment? Nutrients. 2020;12(6):1784. 10.3390/nu12061784 PMC7353365 32560029

[21] Alshwaiyat NM, Ahmad A, Wan Hassan WMR, Al-Jamal HAN. Association between obesity and iron deficiency (Review). Exp Ther Med. 2021;22:1268. 34594405 10.3892/etm.2021.10703 PMC8456489

[22] Ganz T, Nemeth E. Iron homeostasis in host defence and inflammation. Nat Rev Immunol. 2015;15:500–10. 26160612 10.1038/nri3863 PMC4801113

[23] Institute of Medicine. Dietary Reference Intakes for Vitamin A, Vitamin K, Arsenic, Boron, Chromium, Copper, Iodine, Iron, Manganese, Molybdenum, Nickel, Silicon, Vanadium, and Zinc : a Report of the Panel on Micronutrient. Washington; 2001.

[24] WHO/FAO. Vitamin and mineral requirements in human nutrition: report of a Joint FAO/WHO Expert Consultation. 2004;341.

[25] Bresson JL, Burlingame B, Dean T, Fairweather-Tait S, Heinonen M, Hirsch-Ernst KI, et al. Scientific Opinion on Dietary Reference Values for iron. EFSA Journal [Internet]. 2015 [cited 2024 Jul 1];13. Available from: https://www.efsa.europa.eu/en/efsajournal/pub/4254

[26] WHO. WHO guideline on use of ferritin concentrations to assess iron status in individuals and populations. World Health Organization Geneva; 2020. 33909381

[27] Sanders TA, Ellis FR, Dickerson JW. Haematological studies on vegans. Br J Nutr. 1978;40:9–15. 667007 10.1079/bjn19780089

[28] Tong TYN, Key TJ, Gaitskell K, Green TJ, Guo W, Sanders TA, et al. Hematological parameters and prevalence of anemia in white and British Indian vegetarians and nonvegetarians in the UK Biobank. Am J Clin Nutr. 2019;110:461–72. 31190054 10.1093/ajcn/nqz072 PMC6669054

[29] Weikert C, Trefflich I, Menzel J, Obeid R, Longree A, Dierkes J, et al. Vitamin and Mineral Status in a Vegan Diet. Dtsch Arztebl Int. 2020;117:575–82. 33161940 10.3238/arztebl.2020.0575 PMC7779846

[30] Storz MA, Müller A, Niederreiter L, Zimmermann-Klemd AM, Suarez-Alvarez M, Kowarschik S, et al. A cross-sectional study of nutritional status in healthy, young, physically-active German omnivores, vegetarians and vegans reveals adequate vitamin B(12) status in supplemented vegans. Ann Med. 2023;55:2269969. 37851870 10.1080/07853890.2023.2269969 PMC10586079

[31] Bruns A, Nebl J, Jonas W, Hahn A, Schuchardt JP. Nutritional status of flexitarians compared to vegans and omnivores - a cross-sectional pilot study. BMC Nutr. 2023;9:140. 38017527 10.1186/s40795-023-00799-6 PMC10685640

[32] Woo J, Kwok T, Ho SC, Sham A, Lau E. Nutritional status of elderly Chinese vegetarians. Age Ageing. 1998;27:455–61. 9884002 10.1093/ageing/27.4.455

[33] Bruns A, Nebl J, Jonas W, Hahn A, Schuchardt JP. Nutritional status of flexitarians compared to vegans and omnivores - a cross-sectional pilot study. BMC Nutr [Internet]. 2023 [cited 2024 Jul 1];9. Available from:/pmc/articles/PMC10685640 10.1186/s40795-023-00799-6 PMC10685640 38017527

[34] Daru J, Colman K, Stanworth SJ, De La Salle B, Wood EM, Pasricha S-R. Serum ferritin as an indicator of iron status: what do we need to know? Am J Clin Nutr. 2017;106:1634S-1639S. 29070560 10.3945/ajcn.117.155960 PMC5701723

[35] Haider LM, Schwingshackl L, Hoffmann G, Ekmekcioglu C. The effect of vegetarian diets on iron status in adults: A systematic review and meta-analysis. Crit Rev Food Sci Nutr. 2018;58:1359–74. 27880062 10.1080/10408398.2016.1259210

[36] Wang YB, Page AJ, Gill TK, Melaku YA. The association between diet quality, plant-based diets, systemic inflammation, and mortality risk: findings from NHANES. Eur J Nutr. 2023;62:2723–37. 37347305 10.1007/s00394-023-03191-z PMC10468921

[37] García-Maldonado E, Zapatera B, Alcorta A, Vaquero MP. A microalgae docosahexaenoic acid supplement does not modify the influence of sex and diet on iron status in Spanish vegetarians or omnivores: A randomized placebo-controlled crossover study. Nutrition. 2024;118:112282. 38042044 10.1016/j.nut.2023.112282

[38] Slywitch E, Savalli C, Duarte ACG, Escrivão MAMS. Iron Deficiency in Vegetarian and Omnivorous Individuals: Analysis of 1340 Individuals. Nutrients. 2021;13(9):2964. 10.3390/nu13092964 PMC8468774 34578841

[39] Petry N, Egli I, Zeder C, Walczyk T, Hurrell R. Polyphenols and phytic acid contribute to the low iron bioavailability from common beans in young women. J Nutr. 2010;140:1977–82. 20861210 10.3945/jn.110.125369

[40] Armah SM, Boy E, Chen D, Candal P, Reddy MB. Regular Consumption of a High-Phytate Diet Reduces the Inhibitory Effect of Phytate on Nonheme-Iron Absorption in Women with Suboptimal Iron Stores. J Nutr. 2015;145:1735–9. 26041677 10.3945/jn.114.209957

[41] Hunt JR, Roughead ZK. Adaptation of iron absorption in men consuming diets with high or low iron bioavailability. Am J Clin Nutr. 2000;71:94–102. 10617952 10.1093/ajcn/71.1.94

[42] Ambroszkiewicz J, Klemarczyk W, Mazur J, Gajewska J, Rowicka G, Strucińska M, et al. Serum Hepcidin and Soluble Transferrin Receptor in the Assessment of Iron Metabolism in Children on a Vegetarian Diet. Biol Trace Elem Res. 2017;180:182–90. 28342014 10.1007/s12011-017-1003-5 PMC5662660

[43] López-Moreno M, Garcés-Rimón M, Miguel M. Antinutrients: Lectins, goitrogens, phytates and oxalates, friends or foe? J Funct Foods [Internet]. 2022;89:104938. Available from: https://www.sciencedirect.com/science/article/pii/S1756464622000081

[44] Nagessa WB, Chambal B, Macuamule C. Effects of processing methods on phytate and tannin content of black small common beans (Phaseolus vulgaris L.) cultivated in Mozambique. Cogent Food Agric. 2023;9:2289713.

[45] Fukushima A, Uchino G, Akabane T, Aiseki A, Perera I, Hirotsu N. Phytic Acid in Brown Rice Can Be Reduced by Increasing Soaking Temperature. Foods. 2021;10(1):23. 10.3390/foods10010023 PMC7824421 33374851

[46] Pal RS, Bhartiya A, Yadav P, Kant L, Mishra KK, Aditya JP, et al. Effect of dehulling, germination and cooking on nutrients, anti-nutrients, fatty acid composition and antioxidant properties in lentil (Lens culinaris). J Food Sci Technol. 2017;54:909–20. 28303042 10.1007/s13197-016-2351-4 PMC5336446

[47] Egounlety M, Aworh OC. Effect of soaking, dehulling, cooking and fermentation with Rhizopus oligosporus on the oligosaccharides, trypsin inhibitor, phytic acid and tannins of soybean (Glycine max Merr.), cowpea (Vigna unguiculata L. Walp) and groundbean (Macrotyloma geocarpa Ha. J Food Eng. 2003;56:249–54.

[48] Longin CFH, Afzal M, Pfannstiel J, Bertsche U, Melzer T, Ruf A, et al. Mineral and Phytic Acid Content as Well as Phytase Activity in Flours and Breads Made from Different Wheat Species. Int J Mol Sci. 2023;24(3):2770. 10.3390/ijms24032770 PMC9916868 36769092

[49] Feizollahi E, Mirmahdi RS, Zoghi A, Zijlstra RT, Roopesh MS, Vasanthan T. Review of the beneficial and anti-nutritional qualities of phytic acid, and procedures for removing it from food products. Food Res Int. 2021;143:110284. 33992384 10.1016/j.foodres.2021.110284

[50] Smirnoff N. Ascorbic acid metabolism and functions: A comparison of plants and mammals. Free Radic Biol Med. 2018;122:116–29. 29567393 10.1016/j.freeradbiomed.2018.03.033 PMC6191929

[51] Lynch SR, Cook JD. Interaction of vitamin c and iron*. Ann N Y Acad Sci. 1980;355:32–44. 6940487 10.1111/j.1749-6632.1980.tb21325.x

[52] Cook JD, Reddy MB. Effect of ascorbic acid intake on nonheme-iron absorption from a complete diet12. Am J Clin Nutr. 2001;73:93–8. 11124756 10.1093/ajcn/73.1.93

[53] Siegenberg D, Baynes RD, Bothwell TH, Macfarlane BJ, Lamparelli RD, Car NG, et al. Ascorbic acid prevents the dose-dependent inhibitory effects of polyphenols and phytates on nonheme-iron absorption. Am J Clin Nutr. 1991;53:537–41. 1989423 10.1093/ajcn/53.2.537

[54] Yuan G, Sun B, Yuan J, Wang Q. Effects of different cooking methods on health-promoting compounds of broccoli. J Zhejiang Univ Sci B. 2009;10:580–8. 19650196 10.1631/jzus.B0920051 PMC2722699

[55] Fenton HJH. LXXIII.—Oxidation of tartaric acid in presence of iron. J Chem Soc{,} Trans. 1894;65:899–910.

[56] Phaniendra A, Jestadi DB, Periyasamy L. Free radicals: properties, sources, targets, and their implication in various diseases. Indian J Clin Biochem. 2015;30:11–26. 25646037 10.1007/s12291-014-0446-0 PMC4310837

[57] Jomova K, Raptova R, Alomar SY, Alwasel SH, Nepovimova E, Kuca K, et al. Reactive oxygen species, toxicity, oxidative stress, and antioxidants: chronic diseases and aging. Arch Toxicol. 2023;97:2499–574. 37597078 10.1007/s00204-023-03562-9 PMC10475008

[58] Simunkova M, Barbierikova Z, Jomova K, Hudecova L, Lauro P, Alwasel SH, et al. Antioxidant vs. Prooxidant Properties of the Flavonoid, Kaempferol, in the Presence of Cu(II) Ions: A ROS-Scavenging Activity, Fenton Reaction and DNA Damage Study. Int J Mol Sci. 2021;22(4):1619. 10.3390/ijms22041619 PMC7915082 33562744

[59] Weiland A, Wang Y, Wu W, Lan X, Han X, Li Q, et al. Ferroptosis and Its Role in Diverse Brain Diseases. Mol Neurobiol. 2019;56:4880–93. 30406908 10.1007/s12035-018-1403-3 PMC6506411

[60] Bouvard V, Loomis D, Guyton KZ, Grosse Y, Ghissassi F El, Benbrahim-Tallaa L, et al. Carcinogenicity of consumption of red and processed meat. Lancet Oncol. England; 2015; 16(16):1599–600. 10.1016/S1470-2045(15)00444-1 26514947

[61] Bastide NM, Pierre FHF, Corpet DE. Heme Iron from Meat and Risk of Colorectal Cancer: A Meta-analysis and a Review of the Mechanisms Involved. Cancer Prev Res. 2011;4:177–84. 10.1158/1940-6207.CAPR-10-0113 21209396

[62] Sivasubramanian BP, Dave M, Panchal V, Saifa-Bonsu J, Konka S, Noei F, et al. Comprehensive Review of Red Meat Consumption and the Risk of Cancer. Cureus. 2023;15:e45324. 37849565 10.7759/cureus.45324 PMC10577092

[63] Inoue-Choi M, Sinha R, Gierach GL, Ward MH. Red and processed meat, nitrite, and heme iron intakes and postmenopausal breast cancer risk in the NIH-AARP Diet and Health Study. Int J Cancer. 2016;138:1609–18. 26505173 10.1002/ijc.29901 PMC4724256

[64] Genkinger JM, Friberg E, Goldbohm RA, Wolk A. Long-term dietary heme iron and red meat intake in relation to endometrial cancer risk. Am J Clin Nutr. 2012;96:848–54. 22952183 10.3945/ajcn.112.039537

[65] Taunk P, Hecht E, Stolzenberg-Solomon R. Are meat and heme iron intake associated with pancreatic cancer? Results from the NIH-AARP diet and health cohort. Int J Cancer. 2016;138:2172–89. 26666579 10.1002/ijc.29964 PMC4764390

[66] Ward HA, Whitman J, Muller DC, Johansson M, Jakszyn P, Weiderpass E, et al. Haem iron intake and risk of lung cancer in the European Prospective Investigation into Cancer and Nutrition (EPIC) cohort. Eur J Clin Nutr. 2019;73:1122–32. 30337714 10.1038/s41430-018-0271-2 PMC6372073

[67] Aglago EK, Cross AJ, Riboli E, Fedirko V, Hughes DJ, Fournier A, et al. Dietary intake of total, heme and non-heme iron and the risk of colorectal cancer in a European prospective cohort study. Br J Cancer. 2023;128:1529–40. 36759722 10.1038/s41416-023-02164-7 PMC10070394

[68] Huang Y, Cao D, Chen Z, Chen B, Li J, Guo J, et al. Red and processed meat consumption and cancer outcomes: Umbrella review. Food Chem. 2021;356:129697. 33838606 10.1016/j.foodchem.2021.129697

[69] Kuhnle GGC, Story GW, Reda T, Mani AR, Moore KP, Lunn JC, et al. Diet-induced endogenous formation of nitroso compounds in the GI tract. Free Radic Biol Med. 2007;43:1040–7. 17761300 10.1016/j.freeradbiomed.2007.03.011

[70] Gardner CD, Mehta T, Bernstein A, Aronson D. Three Factors That Need to be Addressed More Consistently in Nutrition Studies: “Instead of What?”, “In What Context?”, and “For What?” American J Health Promotion. 2021;35:881–2. 10.1177/08901171211016191d 34120473

[71] López-Moreno M. Comment on Montoro-Garc&iacute;a et al. Beneficial Impact of Pork Dry-Cured Ham Consumption on Blood Pressure and Cardiometabolic Markers in Individuals with Cardiovascular Risk. Nutrients 2022, 14, 298. 10.3390/nu14204266 PMC9611875 36296950

[72] Gaesser GA. Whole Grains, Refined Grains, and Cancer Risk: A Systematic Review of Meta-Analyses of Observational Studies. Nutrients. 2020;12(12):3756. 10.3390/nu12123756 PMC7762239 33297391

[73] Patel L, La Vecchia C, Negri E, Mignozzi S, Augustin LSA, Levi F, et al. Legume intake and cancer risk in a network of case-control studies. Eur J Clin Nutr. 2024;78(5):391-400. 10.1038/s41430-024-01408-w 38321187

[74] Hu J, Wang J, Li Y, Xue K, Kan J. Use of Dietary Fibers in Reducing the Risk of Several Cancer Types: An Umbrella Review. Nutrients. 2023;15(11):2545. 10.3390/nu15112545 PMC10255454 37299507

[75] Bernstein H, Bernstein C. Bile acids as carcinogens in the colon and at other sites in the gastrointestinal system. Exp Biol Med (Maywood). 2023;248:79–89. 36408538 10.1177/15353702221131858 PMC9989147

